# Developing a community-based psycho-social intervention with older people and third sector workers for anxiety and depression: a qualitative study

**DOI:** 10.1186/s12875-017-0648-7

**Published:** 2017-07-12

**Authors:** Tom Kingstone, Heather Burroughs, Bernadette Bartlam, Mo Ray, Janine Proctor, Thomas Shepherd, Peter Bullock, Carolyn Anne Chew-Graham

**Affiliations:** 1 Research Institute for Primary Care & Health Sciences, Keele University, Keele, Staffordshire ST5 5BG UK; 2 South Staffordshire and Shropshire NHS Healthcare Foundation Trust, St. Georges Hospital, Stafford, ST16 3SR UK; 3 School of Health and Social Care, Lincoln University, Brayford Pool, Lincoln, LN6 7TS UK; 4 Age UK North Staffordshire, 83-85 Trinity Street, Hanley, Stoke-on-Trent, ST1 5NA UK; 5 Collaboration for Leadership in Applied Health Research and Care (CLAHRC) West Midlands, Keele University, Keele, ST5 5BG UK

**Keywords:** Older people, Anxiety, Depression, Loss, Third sector

## Abstract

**Background:**

One-in-five people in the UK experience anxiety and/or depression in later life. However, anxiety and depression remain poorly detected in older people, particularly in those with chronic physical ill health. In the UK, a stepped care approach, to manage common mental health problems, is advocated which includes service provision from non-statutory organisations (including third/voluntary sector). However, evidence to support such provision, including the most effective interventions, is limited. The qualitative study reported here constitutes the first phase of a feasibility study which aims to assess whether third sector workers can deliver a psychosocial intervention to older people with anxiety and/or depression. The aim of this qualitative study is to explore the views of older people and third sector workers about anxiety and depression among older people in order to refine an intervention to be delivered by third sector workers.

**Methods:**

Semi-structured interviews with participants recruited through purposive sampling from third sector groups in North Staffordshire. Interviews were digitally recorded with consent, transcribed and analysed using principles of constant comparison.

**Results:**

Nineteen older people and 9 third sector workers were interviewed. Key themes included: multiple forms of loss, mental health as a personal burden to bear, having courage and providing/receiving encouragement, self-worth and the value of group activities, and tensions in existing service provision, including barriers and gaps.

**Conclusions:**

The experience of loss was seen as central to feelings of anxiety and depression among community-dwelling older people. This study contributes to the evidence pointing to the scale and severity of mental health needs for some older people which can arise from multiple forms of loss, and which present a significant challenge to health, social care and third sector services. The findings informed development of a psychosocial intervention and training for third sector workers to deliver the intervention.

## Background

One-in-five older people (65+ years) in the UK experience anxiety and/or depression with prevalence increasing with age [1–5]. Experiences of chronic disease, disability and bereavement have been identified as risk factors for anxiety and depression in later life [6]. Furthermore, anxiety and depression prevalence is reported to be higher among people with co-morbid long term conditions (LTC) [7, 8]. As 60% of people in England aged 60 years and over experience at least one LTC; older people are at risk of developing anxiety and/or depression [9].

Anxiety and depression in older people remain poorly detected and managed [10], particularly for people with chronic physical ill health [11]. If untreated, anxiety and depression may lead to poor quality of life which impacts on increased use of health and social care services and raised mortality [12–14]. Treatment of depression has the potential to improve mortality from all causes in older people [15]. The National Institute for Health and Care Excellence (NICE) guidelines for depression [16] and anxiety [17] advocate a stepped care approach to manage these mental health problems. For mild to moderate symptoms, patients are to be offered advice about lifestyle at step one and low intensity interventions at step two; non-statutory bodies (including third sector, voluntary organisations) may be involved in provision of these services. Task shifting mental health care to community-based programmes has been shown to be effective in low and middle income countries [18, 19]; however, evidence from the UK is limited.

Diagnosis and treatment led by a narrow bio-medical model may overlook important social and contextual factors of mental health, which can inform management [20]. For instance, participation in meaningful activities has an important role in supporting quality of life [21] and mental health and wellbeing in later life [22–25]. Maintaining social participation (social interaction beyond spousal relationship) and meaningful activities (e.g. volunteer roles) have been identified as important following widowhood to support coping [26]. As such, interventions based on increasing participation in meaningful activities have been recommended as a basis for interventions for people with mental health problems, such as depression [27]. Community-based interventions have also been shown to have additional benefits for social inclusion, social cohesion and supporting a sense of belonging and connection [21, 25, 27–32].

The aim of this qualitative study is to explore the views of older people and third sector workers about anxiety and depression among older people in order to refine an intervention to be delivered by Age UK North Staffordshire workers. The qualitative study forms part of a feasibility study, the NOn-Traditional providers to support the management of Elderly People with Anxiety and Depression (NOTEPAD) [33].

## Methods

### Design and sampling strategy

A patient and public involvement and engagement (PPIE) group was established to support the development of this study. The group provided advice on the design of participant-facing documents (e.g. participant information sheet, consent forms) and on the language and terminology used. The PPIE group suggested that the phrases ‘low mood’ and ‘stress’ be used with older people rather than depression and anxiety, respectively, in order to reduce stigma and support engagement. The research team acknowledged that, in particular, ‘stress’ may not be clinically equivalent to anxiety but that there would be sufficient overlap to support exploration. For the remainder of the article the terms ‘low mood’ and ‘stress’ will be used (unless citing the work of others).

This qualitative study explored personal experiences of and perspectives on low mood and stress among older people through semi-structured interviews. A purposive sampling strategy was implemented to recruit two groups of participants: (1) older people (65+ years) with an interest in talking about low mood and stress in later life, and; (2) third sector workers (either working in paid employment or in a voluntary capacity) from organisations providing community-based support to older people, including those with low mood and stress.

### Ethical considerations

Ethical approval was obtained from the Keele University Ethical Review Panel on 23rd September 2015. The article adheres to the Bio Med Central RATS criteria for reporting of qualitative studies (http://old.biomedcentral.com/authors/rats). Recruitment and data collection took place in North Staffordshire between September and November 2015.

### (1) Recruitment of older people

The first and second authors attended group activities (e.g. keep fit, line dancing classes, knitting groups, and luncheon clubs) hosted in North Staffordshire by third sector organisations with which the research team had established links (e.g. Age UK North Staffordshire, Royal Voluntary Service). The researchers explained the study and distributed participant information packs comprising an invitation letter, a participant information sheet, consent to further contact slip and a prepaid envelope. Snowball sampling [34] was used to reach older people who did not attend groups and participant information packs were provided to group attendees to share with friends. Packs were also provided to non-statutory workers to distribute to service users outside of these groups (e.g. befriending, sign-posting services). On return of consent to further contact slips, prospective participants were contacted to confirm willingness to participate and to arrange a convenient time and place for the interview, typically a private room at the community-based venue where group activities were held. Written consent was recorded at the start of the interview.

### (2) Recruitment of third sector workers

Workers were identified through third sector organisations (e.g. Age UK North Staffordshire, Royal Voluntary Service). Worker information packs were distributed via team managers within these organisations and contained equivalent information to the participant information packs for older people (as described). Again, on return of consent to contact slips, prospective participants were contacted to confirm willingness to participate and to arrange a convenient time and place for the interview, typically a private room at their place of work. Written consent was recorded at the start of the interview.

### Data collection

Topic guides were used to semi-structure the interviews with all participants (Table 1):

**Table 1:** Topic guides for semi-structured interviews

	Topic guide for older people	Topic guide for third sector workers
Views on low mood and stress among older people	Explore personal experiences of low mood or stress (or awareness of others who have experienced low mood or stress)	Experience of working with older people with depression and/or anxiety
	Coping and help seeking	Role of third sector organisations in providing support
	Type and effectiveness of healthcare received	Level of training and support for workers
Coping with low mood and stress in the future	Views on experiencing low mood or stress in the future	Views on working with older people with depression or anxiety in the future
	Coping and help seeking in the future	Comfortability and confidence to work with older people with depression or anxiety
	Awareness/appropriateness of Age UK North Staffordshire	Encountering self-harm with older people
Views on the NOTEPAD study	Views on taking part in a research project	Views on training to support older people with depression or anxiety
	Acceptability of one-to-one intervention: visits from third sector workers	Acceptability of one-to-one intervention: training needs and delivery, level of support needed, recording sessions
	Acceptability of group activities	Views on whether older people would find this appropriate
	Concerns or risks	Acceptability and suitability of group activities for depression and/or anxiety

### Data analysis

All interviews were digitally recorded with consent and transcribed by an external company. The study was exploratory in nature; thus, thematic analysis was used to analyse the data [35]. Coding of the transcripts was undertaken by five members of the research team from different professional backgrounds to increase trustworthiness of the analysis [36]. All transcripts were coded inductively by the first and second authors; the third, fourth and last author independently coded five transcripts each. Each data set (older people, third sector workers) was coded separately, and then comparisons across the two data sets were made. Themes emerging from this level of coding were then discussed in team meetings and modified to account for alternative interpretations. The key themes and a model of the analysis were agreed.

## Results

Interviews were completed with 19 older people (18 female; mean age 74 years at point of participation) and 9 third sector workers (8 female; 4 in paid employment, 4 volunteers). The response to the recruitment strategy was low for males and older adults who were not already participating in organised groups (see limitations for further discussion); research data from all participants was included in the analysis.

Analysis of the interview data revealed themes of: multiple forms of loss, mental health as a personal burden, having courage and providing/receiving encouragement, self-worth and the value of groups, and tensions to accessing group activities (including barriers and gaps). Each theme is described below; data is given to support analysis, and each data extract labelled with a participant identifier.

A conceptual model was developed to map older person and third sector worker perspectives on older person responses to loss (a key contributing factor to low mood and stress). The contribution of this analysis to the refinement of the intervention and the NOTEPAD feasibility study is described.

### Experiencing multiple forms of loss

The older people we interviewed described multiple forms of loss in later life, which contributed to feelings of low mood and stress. The forms of loss described included: loss of significant relationships (i.e. spouses, friends, confidantes), changes to physical health and mobility (e.g. osteoarthritis, joint replacement operations), changes in capabilities and established activities (e.g. driving, employment roles), and loss of control of daily life (e.g. burden of providing caregiving).

Typically, older people experienced combinations of losses. For example, one older person described a sequence of losses, which impacted how she dealt with her own low mood:

“My husband died and I had a hip replacement and then three months after, I fell and broke the other [hip]… I’m trying to get over that now but, of course, I’ve had to give my work up, which doesn’t help because you’re more or less stuck in the house 24 hours.” [Older Person 14].

The experience of successive losses contributed to a sense of decline, of being on a “downward slope” [Older Person 6] or in a “downward trend” [Older Person 17].

This experience of loss was typically experienced as a crossroads where the older person found themselves thrown back onto their own resources. They described coming to the realisation that they would have to choose whether to make a conscious effort to get out of the house and forge new social links; or avoid participation and stay indoors. Several interviewees reflected that they feared the latter choice could result in ‘giving up’ and becoming isolated.

Workers recognised the habitual nature of staying indoors:

“A lot of people I see are in their late 80s early 90s they have become used to staying at home, I wouldn’t say they’re house-bound, they’ve got out of the habit of going out.” [Worker 4].

The worker implies an important distinction between older people who are house-bound (i.e. due to physical health impairment) and those who are “out of the habit” of getting out from their home environment. Thus, not going out has become a habitual behaviour for some older people and not due to physical impairment; centralising the importance of behaviour to overcome social isolation and losses attributable to this.

### Distress as a personal burden to bear

One older person described the way in which her friend coped with low mood as: “it was her cross and she would carry it” [Older Person 12]. This metaphor captures common experiences of older people with low mood or stress; as personal burdens for older people to bear. One older person described:

“I had six major operations within about three years. So that really knocked me off my pedestal and nobody really knows… That’s the thing. Depression. Nobody knows what you’re going through… you put on a smile and everybody thinks you’re fine, you’re doing alright, aren’t you? But they don’t realise inside that you’ve been through hell and back and you’re suffering.” [Older Person 10].

The invisibility of such distress, as described, implies an opportunity to control the perceptions of others. A reluctance to share mental health experiences with others was apparent:

“You don't really want to pick up the phone and talk to friends because if you're miserable I'm not going to make her miserable, you know. You've got to do something for yourself.” [Older Person 5].

The reluctance stems from not wanting to burden other people; to share their misery. Some older people did describe sharing these apparent burdens with their General Practitioners (GPs) in order to obtain support; anti-depressant usage was common among participants. However, the problems remained unresolved.

Workers acknowledged how older people did not want to talk about mental health problems, such as anxiety, with them:

“Somehow anxiety has got a capital A, they might talk about feeling a bit low or something, so I think people don’t always find it easy to identify.” [Worker 5].

Older men were commonly identified as a group reluctant to discuss mental health. However, workers described being able to overcome reluctance to talk and perceived stigma about mental health by providing time and developing trust:

“…people do, once they trust you, feel that they can confide and so people have talked about issues around mental health and you listen… quite often it has been enough just to listen but sometimes we would encourage people to have a chat with the GP or maybe direct them towards some counselling.” [Worker 5].

The opportunity to establish meaningful, quality relationships and develop listening skills is important and facilitates encouragement to disclosure to healthcare systems services.

### Having courage and providing/receiving encouragement

Older people described themselves as having to build up courage and determination to participate in group activities despite feelings of low mood and/or stress. One participant described actualising their sense of courage, referring to the time they discovered an advert for a group activity:

“I thought, ‘That sounds good’ but, of course, I hadn’t got the courage because, as I said, [my husband had] only been gone three or four months. Well, I thought, I’ll cut the thing off the [newspaper], which I did, and I popped it into this thing in the kitchen. I had to take it out from time to time and look at it. It took me six months to find the courage to, to phone up.” [Older Person 1].

Courage was important as part of accepting the need to reach out and make social contact. One participant described requiring “grim determination” [Older Person 5]; perhaps indicating how difficult it was to find the necessary courage to engage in a new social activity.

To sustain behaviour change and participation in group activities older people indicated a fear of slipping backwards; sustaining courage and motivation can be challenging as one participant describes:

“…once you drop something it’s so difficult to pick up again afterwards. You think ‘oh, I haven’t been for a long while; what will they think of me if I turn up now?’ you know. But it takes a lot of willpower and not everybody’s got that willpower.” [Older Person 10].

In counter-point to the courage that older people described, workers identified the importance of providing encouragement. Workers expressed empathy towards older people seeking to initiate new social activities, as the following worker describes:

“They need that encouragement to go to the befriending group. I do make contact with everybody a week before to remind them to see ‘do you need a taxi?’, ‘do you need a lift?’” [Worker 4].

The provision of encouragement may act to support older people’s own sense of courage to overcome challenges of low-confidence. Encouragement was reported to be consistently required and reinforced to support joining in.

### Self-worth and the value of group activities

Participation in group activities was valued for several reasons: as an opportunity to develop social relationships, participate in activities and roles, maintain a routine, and also to experience a change of environment. One participant described attending an exercise class as an opportunity for respite from being a caregiver for her husband:

“I don’t work, but this is my day off where I come and enjoy the exercising. I enjoy being with the people… I love my husband, don’t get me wrong, but it’s just nice. He’s in, I can have the car, I can come down here, and it’s just a release sometimes, just to switch off and everything.” [Older Person 16].

Participating in group activities also supported a sense of self-worth within the group, as one participant described: “I really enjoy it and I’m the tea lady” [Older Person 15]. It was clear that participation could also include assuming roles within these groups; others also described providing supportive roles for newcomers.

Workers acknowledged the importance and range of benefits that participating in group activities had for older people. One worker described an exercise group in the following way:

“It isn’t just about sticking the music on and moving your arms and legs, it’s about the interaction with you and the folks in the group and the interaction between each [participant]… just going once a week, say to an exercise group when they have maybe suffered from depression for 2 years and have never been out of the house maybe following a bereavement, it’s more than a lifeline.” [Worker 5].

Acknowledging the role of group activities, beyond the activity itself, is important and has implications for older people to maintain participation, self-confidence and social relationships.

Workers identified that group settings were not appropriate for everyone:

“Even though some of them are still fairly mobile that lack of confidence, I think, of being in a group situation has put them off trying.” [Worker 4].

Providing support and encouragement is important to addressing inertia by overcoming apprehension and lack of confidence regarding group activities.

### Barriers, tensions and gaps in service provision

Internal (intra-personal) and external (inter-personal and systemic) barriers were identified from the experiences of older people. Lack of acceptance and difficulties in acknowledging need (i.e. mental health arising from social isolation or loss of meaningful activities) among older people presented a barrier to engagement with the third sector services; this was evidenced in the continuation of avoidance-style behaviours and social withdrawal. One older person described how she coped with her low mood: “if I feel a bit low, I put the television on” [Older Person 12]. Another shared her views about her friend whom she describes as experiencing low mood:

“She has no hobbies, no interests at all… But she still gets very low and, to my mind, almost enjoys it. That sounds odd, I suppose, but it’s become a way of life and she doesn’t mind saying so.” [Older Person 17].

A lack of opportunities to engage in activities may perpetuate this cycle of social withdrawal; for some of the older person participants, suitable opportunities were considered to be lacking, providing further disincentive.

Transport was identified as a barrier to participation in group activities. Some older people in the study were still able to drive and use their own transport to access these groups; others could no longer drive and described public transport as either unsuitable, or non-existent: “There is no community transport here at all.” [Older Person 9]. Workers acknowledged transport as an issue in the provision of services:

“This is a problem that we have. We know that there are people that are stuck at home… but it's just getting them out and transport is always an issue.” [Worker 3].

Although some workers provided transport to older people, using their own vehicle, the capacity to maintain this raised concerns:

“In the past I might have taken people – picked people up a bit more but we just haven’t got the capacity to do that.” [Worker 6].

Financial cuts to services were described by older people and also workers; this was typically attributed to the policy of fiscal austerity and remained an ongoing concern. Workers would often talk to service users during group activities about financial cuts and service losses, as one older person describes:

“[She’s] always saying they’re cutting back on the company where she works for Age UK, you know. It’s such a shame because a lot of people need these groups.” [Older Person 16].

Where services had been cut, gaps in provision had not been filled and so the needs of those older people who had utilised these services went unmet.

Workers described tensions around the boundaries of their roles as facilitators of group activities (in a paid or voluntary capacity) and responsibility for older people experiencing mental health problems. Workers identified a lack of mental health training:

“We kind of work maybe - try and instinctively to chat to people and reassure them. We don't really know if that’s making much of a difference.” [Worker 6].

Workers had concerns about managing boundaries with older people who presented severe mental health needs:

“You would need to have… good boundaries I think when you’re working with people… so you don't get drawn in to all sorts of things that are going on with that person’s life.” [Worker 6].

This concern was shared by others and reflected a lack of sufficient, tailored training. Workers also identified a tension in the lack of collaboration between third sector and primary care services; important in the context of clients with apparent mental health needs (e.g. older people with dementia, self-harm).

### Conceptual model

The conceptual model is derived from the thematic analysis of the interview data. The experience of loss, as a contributing factor to the mental health needs of older people, is central to the model. How older people respond to this loss varies and is individualised, with courage required in order to accept that a problem exists and to do something about it. Not moving on and not acknowledging a need (i.e. psycho-social needs arising from social isolation or loss of meaningful activities) may then lead to inactivity and increasing social withdrawal. It is this population who are at risk of falling through the cracks of support offered by third sector services.

### Views about the proposed NOTEPAD feasibility study

Overall, older people and third sector workers in this study were positive about the potential for an intervention to be delivered by third sector workers as part of the NOTEPAD study. However, participants reported mixed feelings about whether older people with low mood or stress would take part in the study, specifically group activities:

“If you’ve got a person who wants to sit in the house and not do anything and is not willing to do anything, nothing on this earth will get you out of it.” [Older Person 3].

Older people in the study did not typically associate Age UK North Staffordshire with providing mental health services; this was confirmed by the Age UK North Staffordshire worker interviews. Workers identified training needs, supervision and having the opportunity to de-brief as important in the development of the intervention for the NOTEPAD study:

“You need access to a supervisor and I think depending on when you were having contact with the individuals concerned you would need to be able to de-brief. If you had an urgent concern, you would need to be able to de-brief straight after… you probably also need to be writing something down.” [Worker 5].

Documenting interventions and actions seemed important. About the design of the intervention, the inclusion of one-to-one and group activities was identified as important. Group activities were not seen as suitable for everybody, and one-to-one interventions were considered essential in order to provide opportunity to develop trusting relationships. Furthermore, having a worker accompany an older person to new group settings was considered crucial to providing support and much needed encouragement. Qualitative data that informed study design are summarised in Table 2.

**Table 2:** Qualitative data informing proposed study design

Aspect of study	How intervention has been adapted
Facilitating relationships and trust	• Inclusion of an initial one-to-one appointment between older person and third sector worker at beginning of intervention • Third sector worker to accompany older person to group activity (if required) in order to provide encouragement
Facilitating self-management (and ongoing data gathering)	• Study team have developed an A5 filofax as a resource for older people to support ongoing learning and enable regular recording of reflections on activities
Maintaining participant engagement	• The addition of telephone follow-up appointments with older people – provides flexibility and supports ongoing engagement
Supporting third sector workers	• Providing opportunities to debrief following participant and third sector worker interactions • Training on the maintenance of boundaries between worker and participant • First two intervention sessions will be audio recorded as a means of fidelity checking

## Discussion

**Fig. 1:**
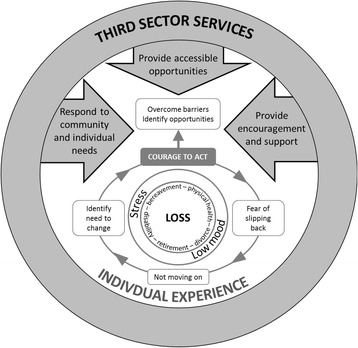
Older people’s and third sector service responses to distress and loss in later life

The themes presented in the results map out how experiences of loss contributed to the mental health of older people in a community sample, their engagement with third sector services, and the provision of these services by those involved in delivery. The process is depicted in the conceptual model (Fig. 1). The experience of loss was described in multiple forms and was a central theme from the interviews. It is clear that loss and its ramifications for older people is a complex and individualised experience; responses to loss, although supported by third sector services, are influenced by biographical factors (e.g. existing support networks, sense of one’s own courage, personal agency). Older people described a reluctance to talk about mental health problems with friends, for fear of burdening them and also experiencing difficulty acknowledging the need to do so; those who self-identified as experiencing low mood or stress would talk to third sector workers once a trusting relationship had been developed. Older people in this study were actively participating in group activities in order to maintain social networks and roles which supported good mental health; older people valued being members of these groups because of the opportunity for social interaction and to support self-confidence. The role of courage and to receive (or provide) encouragement was highlighted as important in supporting this participation; highlighting a key function of third sector workers. Older people revealed an ongoing concern with slipping back to avoidance behaviours; this suggests that maintaining positive behaviours requires ongoing support and encouragement. The findings highlighted important areas for development of the intervention for the NOTEPAD feasibility study and informed: facilitating relationships and trust, facilitating self-management, maintaining participant engagement, and supporting third sector workers.

The data supports other research that identifies the need to take a broader perspective on mental health and its management than the biomedical model permits. For instance, Burroughs et al. [20] described how GPs normalised and justified depression among older people as a social problem reinforced by GP’s sense of powerlessness to respond as they perceived that there were very limited options available to them. In the study reported here, older people identified feelings of distress as a personal burden. Many disclosed anti-depressant usage yet problems remained unresolved and older people in this study took it upon themselves to identify opportunities to attend group activities, which supported their social mental health. Maintaining participation in activities and social interaction seemed important for older people in this study to support quality of life and mental health as identified in other research [21–25] but also to support coping with, and moving on from, the experience of loss. The process of ‘moving on’ has been identified previously in relation to depression [37] in which the role of others (i.e. case managers) was important in providing encouragement. Community-based activities provided by third sector organisations seemed to provide psycho-social benefits for older people in this study, as has been identified elsewhere [21, 27–30, 38, 39]. Chew-Graham et al. [39] described perceived stigma as preventing older people from sharing mental health problems with their GP; this may relate to the fear of burdening others that has been described. In this study an apparent link between loss (in multiple forms) and anxiety and depression was identified; thus, third sector workers may be well placed, with greater contact time, to develop relationships, support disclosure and respond to mental health risk factors. Targeting loss, supporting responses of older people to loss, and overcoming avoidance type behaviours that may lead to rumination, may be a useful point of entry for third sector services.

### Strengths and limitations

This study explored multiple perspectives on anxiety and depression among older people in the community, and identified the central role of loss that contributes to such mental health problems. Interviews were conducted with older people who were already accessing group activities and services in their local community. We did not specifically recruit participants who experienced low mood or stress.

The views from potentially house-bound individuals were limited to those shared by participants about others whom they knew as potentially house-bound. These views may have been shared as a means of supporting social comparisons. This limitation occurred despite efforts from the researchers to recruit more broadly. A male perspective was lacking; this may have been as a consequence of the recruitment strategy which focused on group activities, which were attended in the majority by women. More broadly, this reflects a lack of male-oriented activity groups available in the community.

## Conclusions

This study has identified the central role of loss in later life as potentially contributing to mental health problems; the findings have important implications for the proposed feasibility study, training of the third sector workers delivering the intervention and clinical practice. Third sector workers reported already regular contact with older people presenting apparent mental health problems and needs in the community; typically, such contact occurred at group activities and services not designed to respond to mental health needs, nor with staff or volunteers appropriately trained. With appropriate training third sector workers may be well-placed to deliver a low-level psycho-social intervention to older people, as recommended in the stepped-care approach in NICE guidelines for anxiety and depression, and would provide both older people and primary care with choice for management.

## Abbreviations

LTC: Long Term Conditions
NICE: National Institute for Health and Care Excellence
NOTEPAD: NOn-Traditional providers to support the management of Elderly People with Anxiety and Depression study
PPIE: Patient and Public Involvement and Engagements
